# Integrated Wellbeing: Illustrating the Benefits of Approaching Domain-Specific Development Within an Integrated Framework

**DOI:** 10.3390/healthcare14081086

**Published:** 2026-04-19

**Authors:** Theunis Jacobus De Wet, Tessa De Wet

**Affiliations:** 1 Integrated Development & Employment Alliances (IDEA), Irene, Pretoria 0157, South Africa; 2 Optentia Research Unit, Faculty of Humanities, North-West University, Vanderbijlpark 1900, South Africa

**Keywords:** integrated wellbeing, financial wellbeing, physical wellbeing, Cobb–Douglas model, lifespan, health span, systemic optimisation, resource allocation

## Abstract

**Background:** Human wellbeing consists of dynamic interactions and feedback loops across multiple life domains, a perspective increasingly emphasised within positive psychology’s systemic and strengths-based approach to flourishing. This study develops a systemic framework to model these interdependencies and examines how cross-domain investment can optimise both domain-specific and integrated wellbeing across the lifespan. **Methods:** Using a Cobb–Douglas functional form with associated growth and resource constraints, we formalise the interaction between physical and financial wellbeing as an example and analyse their joint contribution to overall wellbeing. **Results:** The model demonstrates that improvements in one domain of wellbeing can enhance wellbeing in another, thereby shifting the optimisation frontier. While narrow domain-specific wellbeing strategies are subject to larger diminishing marginal returns, cross-domain investment generates reinforcing effects that elevate both domains simultaneously and increase integrated wellbeing. **Conclusions:** In line with positive psychology’s focus on leveraging strengths to support areas of relative weakness, the findings show how developing one domain of wellbeing can mitigate constraints in another. These findings align with positive psychology’s emphasis on multidimensional flourishing and resource-building processes, highlighting the importance of systemic resource allocation and suggesting that wellbeing optimisation requires coordinated, contextualised multi-domain strategies rather than siloed approaches.

## 1. Introduction

Definitions and descriptions in the human wellbeing (WB) domain are evolving as understanding of WB expands to include psychological, social, behavioural, and existential dimensions. Positive psychology (PP) has increasingly emphasised flourishing rather than merely the absence of illness. Contemporary third-wave PP highlights systemic complexity and the dynamic interaction of life domains [1], while multidimensional models of wellbeing conceptualise flourishing as the integration of different dimensions of human behaviour [2]. However, few quantitative frameworks exist that model this systemic dynamic in formalised mathematical models that explicitly integrate cross-domain wellbeing dynamics.

Consistent with this systemic orientation, this study expresses an individual’s wellbeing as a function of the different wellbeing domains. Given the characteristics of wellbeing and the domains thereof, a Cobb–Douglas functional form is used as the mathematical expression. This functional form expresses a resulting outcome as a smooth, multiplicative combination of inputs, where each input contributes positively but with diminishing marginal impact, and inputs can partially substitute for one another [3]. It is useful in the wellbeing context as it allows one to express how each wellbeing domain contributes to overall wellbeing and how the different domains interact to increase or limit their contributions to overall wellbeing.

While we do not provide statistical estimates of the elasticities for the function, the Cobb–Douglas formulation offers a transparent and theoretically grounded framework for organising multidimensional wellbeing and clarifying assumptions regarding interaction effects across the life course. Importantly, it contributes to the literature that addresses a key gap in the wellbeing literature: the absence of a unified structural representation linking multiple domains within a single formal model. By moving beyond descriptive indices and siloed empirical correlations, the approach contributes analytical coherence, theoretical integration, and a platform for future empirical and dynamic extensions in interdisciplinary wellbeing research.

We show how this approach can lead to analytically coherent conclusions and insights by using the framework in a two-factor model that expresses human wellbeing as a function of financial and physical wellbeing. We focus on these two factors to express the usefulness of the framework because of the relatively high degree of agreement on how each of these factors contribute to human wellbeing. It also allows for a clear distinction between health span and lifespan which differentiates the framework from standard financial lifecycle models. The results emphasise the importance of considering other domains of wellbeing when optimising for a single domain or overall wellbeing. For example, using the framework we highlight that financial wellbeing can increase by improving physical wellbeing. This improvement is achieved through improved ability to earn income but also by lowering the required return that must be achieved on financial wealth. With improved physical and financial wellbeing, the individual’s overall wellbeing also increases.

In the following sections the concept literature is provided, after which the analytical framework for iWB is presented, followed by a description of the specific functions for analysing financial and physical wellbeing. Propositions and insights based on the framework and functions are then provided, ending with a discussion including future research.

## 2. Literature Review

### 2.1. Integrated Wellbeing (iWB)

Integrated wellbeing is a systems-based, multi-domain framework which is used for understanding human flourishing, where biological health, psychological functioning, social connection, economic stability, environmental conditions, and personal meaning interact (non-linearly) to determine life outcomes [4,5,6,7]. It is often used in health span economics, organisational wellbeing programmes, and capability-based models such as Sen’s and Nussbaum’s approaches [8,9]. Integrated wellbeing emphasises:

1. Multidimensionality: Wellbeing spans many life domains;
2. Integration: The domains affect each other;
3. Sustainability across the life course: Wellbeing is dynamic and adapts as circumstances and contexts change;
4. Agency and capability: It is the individual’s ability to act, adapt, and make choices that support flourishing;
5. Contextual alignment: It is dependent on the goals, the environment, and available resources of both the individual and the society in which they find themselves.

Integrated wellbeing matters on a global and national level because an improvement in wellbeing can increase performance and productivity, leads to better employment outcomes, affects stronger social cohesion, and maintains sustainable growth [7]. The opposite is also true. Decreases in any area of the wellbeing domain would decrease these on micro and macro levels. Another consideration is that there is also growing dissatisfaction with current measures of economic progress (such as the use of GDP growth) [10].

On an individual level integrated wellbeing matters because it determines how long and well you live, how you work, earn, and adapt to changes over the lifespan [7].

The literature on integrated wellbeing identifies several domains that are important for human flourishing. Although the extent of the domains that have been identified varies depending on the level of investigation (e.g., global, national, local, individual) or the detail that the investigation requires, there are a few domains that surface in almost all the studies. These are: mental wellbeing; occupational wellbeing; financial wellbeing; physical wellbeing; social wellbeing; and community wellbeing. In fact, Rath et al. explored the common elements of wellbeing that transcend countries and cultures and concluded that these five domains are indeed statistically distinct factors that impact individual wellbeing [11].

While it is essential to understand each domain that impacts wellbeing, examining the different domains of wellbeing through an integrated framework is essential because these components do not operate independently in lived experience or in socio-economic systems.

Mental, physical, financial, occupational, and social wellbeing interact continuously, reinforcing or constraining one another over time. For example, physical health influences productivity and earnings capacity; financial stability affects stress levels and mental health; occupational engagement shapes social networks and purpose; and community cohesion influences both safety and opportunity. Analysing these domains in isolation risks overlooking feedback effects, compounding advantages, and cumulative vulnerabilities that shape life-course outcomes. An integrated perspective therefore allows for a more realistic understanding of how wellbeing is produced, sustained, or eroded, at both the individual and societal levels. By recognising interdependence rather than fragmentation, such a framework contributes to clearer policy design, more accurate diagnosis of systemic weaknesses, and a deeper theoretical understanding of human flourishing across contexts.

The integrated nature of the different domains of wellbeing suggests that:

1. An improvement in an individual’s total wellbeing will be limited if only one domain is developed perpetually and in isolation of the other domains. It implies that each domain is subject to diminishing marginal returns in relation to overall wellbeing. For example, while an improvement in physical wellbeing from a low level should initially contribute significantly to a lift in integrated wellbeing, it is unlikely to continue to increase integrated wellbeing if other domains, such as occupational wellbeing, remain neglected. Kahneman et al. indicate this in the context of financial wellbeing by noting that an individual’s (subjective) wellbeing initially lifts along with their income [12]. However, at a certain level the impact starts to taper off and their wellbeing becomes constrained by other domains.
2. The development of one domain has an impact on the other domains. Some domains may have a significant impact while others have a smaller impact, which may be dependent on the context the individual finds themselves in. For example, an improved level of physical wellbeing will have an impact on career wellbeing, while an improvement in career wellbeing has an impact on financial wellbeing.
3. This also suggests that there may be sacrifices (a “budget and/or resource constraint”) involved in one domain to develop and maintain sustained integrated wellbeing over the lifespan. An individual has only limited resources available that they gain from one domain to develop another domain, and it may be necessary to sacrifice gains in one domain in the short term to develop another domain.

The characteristics described above make the Cobb–Douglas production function a particularly appropriate functional form for expressing integrated wellbeing. First, its multiplicative structure reflects multidimensionality and interdependence by modelling overall wellbeing as jointly produced by several distinct but interacting domains. Second, the functional form naturally embeds diminishing marginal contributions within each domain, aligning with the observation that continuous development of a single domain in isolation yields progressively smaller gains in total wellbeing. Third, because each input contributes proportionally rather than independently, the specification captures complementarity: deficiencies in one domain constrain the returns from improvements in others. This is consistent with the life-course dynamics described above, where physical, occupational, financial, and social wellbeing reinforce or limit one another. Finally, the Cobb–Douglas framework accommodates resource constraints, as individuals allocate limited time, energy, and financial resources across domains in pursuit of sustained flourishing. By translating these conceptual features into a coherent analytical structure, the model provides theoretical discipline, internal consistency, and a transparent basis for examining trade-offs and integration in human wellbeing.

Although not always explicitly expressed as Cobb–Douglas functional formulations, the functional form has often been used in health economics, healthcare production and the broader wellbeing literature. For example, Gupta et al. [13] estimate the impact of public spending and governance on health outcomes using models reflecting Cobb–Douglas specifications while Joumard et al. [14] model health system performance using a production function approach. Dieleman et al. [15] linked national health spending and system resources to health performance using nonlinear, multi-input empirical specifications that are consistent with a Cobb–Douglas-type framework.

While alternative functional forms could be used to model integrated wellbeing, each with distinct implications, the Cobb–Douglas functional form provides a balanced approach that captures complementarity and diminishes marginal contributions while maintaining analytical tractability and theoretical parsimony. This makes it well suited for expressing the integrated yet resource-constrained nature of human wellbeing without imposing unnecessary structural rigidity. Two alternative functional forms that can be considered are the more flexible CES or translog functions which allow for varying degrees of substitutability. They do, however, introduce additional complexity and parameter requirements that are difficult to justify in a primarily conceptual framework.

### 2.2. Two Domains of iWB as Illustration

To illustrate how this system and its interdependencies can be utilised for integrated as well as domain-specific optimisations, we narrow the scope of all the integrated wellbeing domains to only two domains:

1. Financial wellbeing (F);
2. Physical wellbeing (P).

While it is clearly an over-simplification of the complex reality of integrated wellbeing, this simplification allows us to illustrate:

1. How the interdependence between the factors of wellbeing should be considered when addressing single domains—in this case, financial and physical wellbeing;
2. How considering a holistic approach of wellbeing (integrated wellbeing) can also contribute to optimisation of the overall integrated wellbeing of the individual.

We start the illustration with a brief description of each of these two domains within the context of the integrated wellbeing framework.

#### 2.2.1. Financial Wellbeing

While different definitions of financial wellbeing exist, its contribution to overall wellbeing is well established. According to Netemeyer et al., financial wellbeing explains “*substantial variation in well-being beyond other life domains that have been the focus of prior research (*i.e.*, job satisfaction, relationship support satisfaction, and physical health*” [16]. In short, it is an integral part of wellbeing (integrated wellbeing).

Riitsalu et al. note that there are many terms used for financial wellbeing [17]. These include financial wellness, financial health, financial satisfaction, financial comfort and financial resilience. Bruggen et al. highlight that the existing definitions and measures can be clustered into three groups in terms of their approach: those that use objective and subjective characteristics, and those that use either objective or subjective measures of financial wellbeing [18].

Examples of subjective definitions include a 2021 United Nations report in which it is stated that “*Financial health—or wellbeing—is an emerging concept that addresses the financial side of individuals’ and families’ ability to thrive in society*” [19]. Similarly, Bruggen et al. define financial wellbeing as “*the perception of being able to sustain current and anticipated desired living standards and financial freedom*” [18]. Netemeyer et al. describe financial wellbeing as the result of two distinct yet related assessments: “*How am I doing today” and “How do I expect I will be doing in the future*?” [16].

Definitions presented by Vosloo et al. and Cox et al. are examples of definitions that combine both objective and subjective aspects [20,21]. Vosloo et al. define financial wellbeing as “*objective and subjective aspects that contribute to a person’s assessment of their current financial situation*”, while Cox et al. define financial wellbeing as “*a composite indicator made up of objective and subjective dimensions and concepts which describe individuals’ financial state and financial behaviours*”. The Consumer Protection Bureau defines financial wellbeing as “*a state of being wherein a person can fully meet current and ongoing financial obligations, can feel secure in their financial future, and is able to make choices to enjoy life*” [22].

Objective measures of financial wellbeing include levels of debt, income or financial ratios such as debt-to-income levels. An example of a study that includes these measures is Greninger et al. [23].

Financial wellbeing also impacts other domains of integrated wellbeing. Bruggen et al. highlight that financial wellbeing has a wide-ranging and consequential impact on enablement of individuals, organisations and societies [18]. On an individual level, financial wellbeing impacts the quality of life, success, happiness, general wellbeing, mental health and relationship quality. Low financial wellbeing that leads to financial strain can have a negative impact on physical wellbeing. For example, Mercado et al. show that lower financial wellbeing is associated with unfavourable health behaviours and physical and mental health conditions [24].

Apart from physical wellbeing, it also impacts career wellbeing and social wellbeing. For example, at the organisational level, employees with low financial wellbeing tend to be more absent from work and have lower productivity which could impede their work and performance [20,25]. At a societal level Bruggen et al. state that large groups of people that face financial problems reduce consumption and require more social (welfare) support [18].

#### 2.2.2. Physical Wellbeing

Despite ongoing work in different disciplines concerning how to define physical wellbeing, its importance for integrated wellbeing is unambiguous. Physical wellbeing is negatively correlated with mortality risk, and positively with physical functioning and independence and cognitive ability [26,27].

As with financial wellbeing, there are several definitions and descriptions of physical wellbeing. Pressman et al. describe the different approaches at defining physical wellbeing [28]. They suggest that physical health researchers tend to refer to physical wellbeing as the absence of disease, while other models of physical wellbeing view it through a continuum which anchors one side of the continuum to disease, disability and death and the other side to optimal human functioning where “a person is maximally healthy, has resources for resisting disease and is capable of experiencing joy”.

The notion that physical wellbeing is a domain that goes beyond just objective measures of health, is supported by the description from the World Health Organisation which defined health in its constitution as “*a state of complete physical, mental and social wellbeing and not merely the absence of disease or infirmity*” [29]. Diener et al. define physical wellbeing as “*the condition in which an individual experiences good physical health, sufficient energy and physical capability to engage fully in life activities and pursue valued goals*.” [30]. Rath et al. define physical wellbeing as having good health and enough energy to get things done daily [11].

In short, an improvement (or decrease) in physical wellbeing not only increases (or decreases) a person’s lifespan, but it increases (or decreases) the potential of a person living a life free from (saddled with) activated/triggered chronic disease and fragility. Not surprisingly, it also affects an individual’s ability to build savings and earn income for a longer period [25].

Physical wellbeing impacts the other domains of integrated wellbeing. For example, Pintor et al. reviewed the literature that investigates the relationship between an individual’s health and their associated labour market outcomes [26]. “*The evidence reviewed suggested that individuals with better health overwhelmingly exhibit higher earnings and often enhanced labour supply*.” The improvement in earnings is linked to higher productivity, which leads to increased earnings per unit of labour supplied. The lift in productivity is associated with enhanced physical and mental capabilities that are associated with improved physical wellbeing.

Higher earnings can also be associated with higher labour supply. That said, this mechanism depends on an individual’s preference between work and leisure. Improved physical wellbeing can result in an individual choosing to increase their labour supply, while the opposite can also be true [31]. There does, however, seem to be consensus among researchers that poor health reduces labour supply [27]. It is worth noting that a growing body of research suggests that working longer increases a person’s standard of living in retirement which can be another factor that contributes to an individual’s decision to provide more labour [28,32].

While there seems sufficient evidence that suggests that an individual’s ability (capability) to increase their labour supply improves with an improvement in physical wellbeing [33], this does not necessarily imply that they will indeed lift their supply [34]. To illustrate the positive impact that an increase can have on an individual’s wellbeing we assume they do indeed decide to translate the capability to increase supply into an actual increase.

Another channel through which physical wellbeing can have an impact on an individual’s savings and earnings (financial wellbeing) is by means of the costs associated with a deterioration in physical wellbeing (as measured through health expenditure). For example, it can have a negative impact on savings if a deterioration results in increased medical expenses [35].

Finally, for the purposes of the development of our analytical model, it is important to distinguish between healthspan and lifespan when considering physical health as it has direct bearing on describing the period during which a person can contribute productively in its working environment (health span) and the period in which an individual lives (lifespan) [36].

In the following section we use the defining background on integrated wellbeing, financial wellbeing, and physical wellbeing to express these as a dynamic interrelated system that should be optimised within an individual’s respective resource constraints.

## 3. A General Framework for Analysing Integrated Wellbeing

We first present a general analytical framework that can be used to optimise integrated wellbeing across several wellbeing domains. Table 1 provides a description of the variables utilised. Once the framework is established, we narrow it down to the two domains—physical and financial wellbeing—to illustrate its use.

As noted, a Cobb–Douglas function is utilised to express the relationship between the different domains and integrated wellbeing (IW). The following characteristics of integrated wellbeing render a Cobb–Douglas functional form, particularly useful for:

- The complimentary nature of the different domains of integrated wellbeing;
- The positive elasticities associated with each domain of integrated wellbeing towards overall wellbeing;
- The diminishing marginal contribution of each single domain towards overall integrated wellbeing;
- The resource constraint that restricts the extent to which each domain can be developed during a certain period.

While it is not the intent of this paper to provide statistical estimates for the relevant elasticities, if estimated, the absolute and relative magnitudes of the elasticities obtained from a Cobb–Douglas estimation hold important consequences for individual decision-making. These include:

- The elasticities associated with each wellbeing domain represent the percentage change in overall wellbeing in response to a one percent change in the specific wellbeing domain. This allows the individual to gauge where his/her marginal effort will result in the highest marginal gain in integrated wellbeing.
- For given elasticities, marginal gains are larger when a domain is low.
- However, over-investment in one domain of wellbeing yields diminishing marginal gains in integrated wellbeing.

Beyond the Cobb–Douglas presentation of integrated wellbeing, we expand the framework by adding a dynamic function for the development of each domain and a function that represents the resource constraint(s).

### 3.1. The Cobb–Douglas Presentation of Integrated Wellbeing

The Cobb–Douglas presentation of the integrated wellbeing function (*IW*) for time (t) is expressed as:

(1) $${IW}_{t}=\;\prod_{i=1}^{n}x_{i,t}^{\theta_{i}}\;\theta_{i}>0$$

where

$$\sum_{i=1}^{n}\theta_{i}=1$$

${IW}_{t}$ is an index that represents the integrated wellbeing of an individual at time (t): a value approaching zero represents a very low level of integrated wellbeing and a value approaching 1 represents a very high level of integrated wellbeing.

$x_{i,t}\;$are indices that represent different levels of wellbeing of domain (i) at time (t) (i = 1 to n). A level approaching 0 represents a very low level of domain-specific wellbeing and a level approaching 1 represents a very high level of domain-specific wellbeing.

$\theta_{i}$ represents the elasticity of wellbeing domain i on integrated wellbeing. These elasticities show the impact (in percentage terms) of a one percent change in a wellbeing domain i on integrated wellbeing. We assume constant returns to scale as a normalisation property.

### 3.2. Progression of Each Domain of Integrated Wellbeing Within the Individual’s Resource Constraints

Each domain requires resource expenditure to maintain and develop it over time. Resource allocation to domain i affects $x_{i,t}$ through the investment function $\left(I_{i}\right)$:

(2) $$I_{i,t}=\;I_{i}(C_{i,t})$$

where

$C_{i,t}$ represents total spending on domain i.

$$\begin{array}{c} C_{i,t}\geq 0;\\ I_{i,t}\geq 0,\;I_{i}^{\prime }\text{>}\;0,\;I_{i}^{\prime \prime }<0 \end{array}$$

We also control for the possibility that the level of wellbeing of a domain can decline due to natural causes (such as the impact of old age on physical wellbeing), or due to using the domain to develop other domains (such as the expense of funds to improve physical health).

Taking into account the above we express the progress function for wellbeing domain i as:

$$x_{i,t}=\;min\;\left\{1,\;\left(1-\delta_{i}\right)x_{i,\;t-1}+\;I_{i}(C_{i,t})\right\}$$

where

$\delta_{i}\in [0,1]$ represents the decay rate of wellbeing domain i.

However, there is a finite level of resources that can be allocated to develop other domains. This represents the individual’s wellbeing resource constraint ($R_{t}$). We also assume a no-borrowing constraint.

$$\sum_{i=1}^{n}C_{i,t}\leq R_{t}$$

where

$$C_{i,t}\geq 0$$

We assume that investment into wellbeing domain i, and the resources expended on such investment cannot be negative.

The function that generates resources for investment across wellbeing domains in period (t + 1) is a function of:

- The available resources at the end of the previous period $R_{t}$;
- The ability to create new resources based on the domains entering the integrated wellbeing function

(3) $$R_{t+1}=R_{t}+Y\left(x_{1,t},\ldots ,x_{n,t}\right)-\sum_{i=1}^{n}C_{i,t}$$

where

$$\frac{\partial Y}{\partial x_{j,t}}>0\;\text{for relevant j.}$$

We can use this system to optimise integrated wellbeing subject to the individual’s level of resources available to develop the different domains. The system (variables in Table 1) also allows us to surface the impact of changes in individual wellbeing domains on other wellbeing domains and overall wellbeing (integrated wellbeing).

## 4. Applying the Framework to Physical and Financial Wellbeing

### 4.1. Optimising Integrated Wellbeing in a Two-Domain Model That Includes Financial and Physical Wellbeing

We can now adopt the system described in Section 3 into a two-factor model that includes financial and physical wellbeing as drivers of integrated wellbeing. This illustrates the broader principle that consideration of all the wellbeing domains can provide additional optionality when focusing on a domain-specific outcome.

And, in this specific example, it illustrates how the consideration of integrated wellbeing when optimising financial wellbeing can contribute to a more optimal financial wellbeing outcome, while also leading to a better physical and integrated wellbeing outcome and vice versa—an improvement in financial wellbeing can contribute to an improvement in physical wellbeing. The system below describes the dynamics for each of these wellbeing domains within the integrated wellbeing framework.

### 4.2. The Integrated Wellbeing Function

Making the simplifying assumptions that the only domains entering the integrated wellbeing function are financial and physical wellbeing, we can express the integrated wellbeing function of an individual at time (t) as:

(4) $${IW}_{t}=\;F_{t}^{\theta_{F}}P_{t}^{\theta_{P}}$$

where

$\theta_{F}\;and\;\theta_{P}$ represent the elasticity of financial wellbeing and physical wellbeing respectively and:

$$\theta_{F},\theta_{P}>0$$

$$\theta_{F}+\theta_{P}=1$$

$F_{t}$ $\in \left[0,1\right]$ is an index of financial wellbeing in time t (For analytical clarity and comparability across domains measured in different units, financial and physical wellbeing are normalised to lie between zero and one, allowing the integrated wellbeing function to be interpreted as a bounded index rather than a scale-dependent output measure. The indexation should be applied for any wellbeing domain that enters the integrated wellbeing function when the approach is empirically applied).

$P_{t}$ $\in \left[0,1\right]$ is an index of physical wellbeing in time t, where a value approaching 0 represents very poor financial and/or physical wellbeing while a value approaching 1 represents an exceptional level of financial and/or physical wellbeing.

The financial wellbeing index is a function of objective financial wellbeing (O$F_{t}$), which is measured in currency value and represents the individual’s financial wealth as a stock, and a subjective component of financial wellbeing (${SF}_{t}$), which represents an index of the individual’s perception of their level of financial wellbeing. The index is positively correlated to both objective and subjective financial wellbeing and remains bounded by 0 and 1:

(5) $$F_{t}=f_{F}\left({{OF}_{t};SF}_{t}\right),$$

where

$$SF_{t}\in \left[0,1\right],\;f_{F}^{\prime }\left({OF}_{t}\right)>0,\;{\;f}_{F}^{\prime }\left({SF}_{t}\right)>0$$

and

$$f_{F}:R_{+}\times \left[0,1\right]\to [0,1]$$

For illustrative purposes we use Diener et al.’s definition of physical wellbeing and assume the index represents “*the condition in which an individual experiences good physical health, sufficient energy and physical capability to engage fully in life activities and pursue valued goals*”.

### 4.3. The Progression of Physical Wellbeing

The individual’s physical wellbeing in time t + 1 develops as a function of physical wellbeing $P_{t}$ and health investment, $I_{p}(C_{OF,t})$, where $C_{OF,t}$ is health-related spending out of objective wealth. This can be viewed as the use of financial resources to access health care, fitness centres, healthy food, etc.

The progression of the physical wellbeing domain is bounded by the unit interval and expressed as:

(6) $$P_{t+1}=\min\left\{1,\;(1-\alpha_{t})P_{t}+I_{P}\left(C_{OF,t}\right)\right\}$$

$$P_{t}\in [0,\;1],\;C_{OF,t}\geq 0,\;{\;I}_{P}^{\prime }\left(C_{OF,t}\right)>0$$

where

$I_{P}$ represents investment in physical wellbeing.

$C_{OF,t}$ represents objective financial resources spent on improving physical wellbeing.

$\alpha_{t}$ represents the rate of decay of physical wellbeing through time. This rate of decay is best expressed through a bounded logistic transition function which allows α to evolve smoothly over time (t).

(7) $$\alpha_{t}=\alpha_{min}+\frac{\alpha_{max}-\alpha_{min}}{1+exp\left[-\tau (t-t_{0})\right]}\;\mathrm{and}\;0<\alpha_{min}<\alpha_{max}<1$$

where

$\alpha_{min}$ represents the lowest natural rate of physical decline for the individual. This typically corresponds with early adulthood.

$\alpha_{max}$ represents the highest natural rate of physical deterioration for the individual. This typically corresponds to late-life accelerated ageing

τ represents the speed of transition between low to high rate of physical decline for the individual.

$t_{0}$ represents the inflexion point where the rate of acceleration is the fastest.

Apart from recognising the decay rate of physical wellbeing, we also distinguish between two periods in the individual’s lifetime—the individual’s health span and lifespan:

- Health span $H_{t},$ is defined as the expected remaining time that they are physically able to contribute productively to society and still earn an income by providing labour;
- Lifespan $T_{t}$ is defined as the individual’s expected remaining living years (up to death) where
  $$H_{t}\geq 0,\;\mathrm{and}\;T_{t}\geq 0;$$
  $$H_{t}\leq T_{t}$$

The individual’s health span and lifespan are positively related to physical wellbeing, but the impact of physical wellbeing is larger on health span than on lifespan.

(8) $$H_{t}=h(P_{t})$$

(9) $$T_{t}=g(P_{t});$$

where

We assume that ${h(P}_{t})$ is continuous and non-negative, with $h^{\prime }\left(P_{t}\right)>0$, and that there exists a threshold level of physical wellbeing ${\bar{P}}_{H}\in \left[0,1\right]$ such that:

$${h(P}_{t})=\;0\;\mathrm{for}\;P_{t}\leq {\bar{P}}_{H}$$

$$h^{\prime }\left(P_{t}\right)>g^{\prime }\left(P_{t}\right)\geq 0\;\mathrm{for}\;\mathrm{all}\;\mathrm{relevant}\;P_{t}.$$

This distinction between lifespan and health span is an important factor that contributes to improved understanding of how physical and financial wellbeing interact positively to improve each other and impact on an individual’s overall integrated wellbeing.

### 4.4. The Progression of Financial Wellbeing

The individual’s financial wellbeing in time (t) progresses as its objective financial wellbeing (${OF}_{t}$) improves. Objective financial wellbeing increases according with the individual’s financial wealth which is explained in this section. We assume objective financial wealth is liquid and can be allocated between expenditure on physical wellbeing and financial investment.

### 4.5. The Resource Generation and Constraint Functions

The resources available to allocate to investment into physical and financial wellbeing is a function of:

- The individual’s previous level of resources (${OF}_{t-1}$);
- The application of physical wellbeing in the pursuit of earning income and financial resources (OF) in the pursuit of financial resource earnings:
  $$G_{R}\left(P_{t-1},{OF}_{t-1}\right).$$

The generation of resources to use for investment into physical and financial wellbeing takes the form of labour income, $Y(P_{t-1})$ that the individual generated with their physical wellbeing in the previous period and interest income ($r_{t-1}$) that the individual earned on his/her objective financial wealth in the previous period (O$F_{t-1}$).

The resource generating function (resource flow) can therefore be expressed as:

(10) $$G_{R}\left(P_{t-1,\;\;}{OF}_{t-1}\right)=Y\left(P_{t-1}\right)+\;r_{t-1}{OF}_{t-1}$$

where

$$Y\left(P_{t-1}\right)=\left\{\begin{array}{c} Y\left(P_{t-1}\right),\;H_{t-1}>0\\ 0,\;\;H_{t-1}\leq 0 \end{array}\right.$$

Which creates the explicit constraint that an individual can earn labour income from her/his physical wellbeing during his/her health span and only financial income from her/his objective financial wealth thereafter.

In our two-domain wellbeing model, the available resources in time t, ${OF}_{t}$, are spent on investment in physical wellbeing (I$\left({OF}_{P,t}\right))$ and investment in financial wellbeing ($I\left({OF}_{t}\right)).$ Because of the “no-borrowing” assumption, it implies that the spending on physical and financial wellbeing is constrained by the available resources in time t. This constraint is expressed as:

(11) $${OF}_{t-1}+G_{R}\left(P_{t-1},{OF}_{t-1}\right)\;\geq \left[C_{OF,t-1}+\;I_{OF,t-1}\right]$$

The individual’s financial resources to allocate to investment in financial wellbeing at time t is expressed as:

(12) $${OF}_{t}=\;{{OF}_{t-1}+r}_{t-1}OF_{t-1}+{Y(P}_{t-1})-C_{OF}\mathrm{if}\;H_{t-1}>0$$

$${OF}_{t}={OF}_{t-1}+r_{t-1}OF_{t-1}-C_{OF}\mathrm{if}\;H_{t-1}\leq 0$$

We can now use this framework to deduce a few insights related to the optimising of integrated wellbeing, financial wellbeing and physical wellbeing.

## 5. Propositions from an Integrated Wellbeing Perspective

This system of integrated wellbeing illustrates how the interconnected nature of wellbeing domains can be used to optimise a specific domain while simultaneously improving overall wellbeing. We use the framework to deduce a few propositions around financial, physical and integrated wellbeing.

**Proposition** **1.** *In the two-domain case, financial resources allocated to physical wellbeing possess an intertemporal option value: by preventing physical wellbeing from falling below the health span threshold, such investment preserves the individual’s capacity to generate labour income. This preserved earning capacity supports future wealth accumulation and, through the financial wellbeing function, strengthens financial wellbeing over the lifespan. We deduct it through the following steps:*

1. Health spending increases physical wellbeing (from Equation (6)):

$$P_{t+1}=\prod_{\left[0,1\right]}\left[(1-\alpha_{t})P_{t}+I_{P}\left(C_{OF,t}\right)\right]$$

with

$$I_{P}^{\prime }\left(C_{OF,t}\right)>0;$$

therefore

$$\frac{\partial P_{t+1}}{\partial C_{OF,t}}>0$$

2. Physical wellbeing determines health span (from Equation (8)):

$$H_{t}=h(P_{t})$$

with

$$h^{\prime }\left(P_{t}\right)>0$$

and a threshold $\bar{P_{H}}$ exists—such that

$$h\left(P_{t}\right)=0\mathrm{for}\;P_{t}<\bar{P_{H}};$$

therefore, higher $P_{t}$ reduces the probability of entering the regime $H_{t}=0$.

3. Labour income depends on health span (from Equation (10)):

$$Y\left(P_{t-1}\right)=\left\{\begin{array}{c} Y\left(P_{t-1}\right),\;H_{t-1}>0\\ 0,\;\;{\;H}_{t-1}\leq 0; \end{array}\right.$$

therefore, maintaining $H_{t-1}>0$ preserves positive labour income.

Crossing this threshold eliminates labour income entirely.

4. Labour income increases wealth (from Equation (12)):

$${OF}_{t}={{OF}_{t-1}+r}_{t-1}OF_{t-1}+{Y(P}_{t-1})+I_{OF,t}-C_{OF,t}\mathrm{if}\;H_{t-1}>0$$

where an increase in ${Y(P}_{t-1})$ increases wealth accumulation.

If ${\;H}_{t-1}$ = 0, the term disappears; therefore, preserving labour income raises the future path of ${OF}_{t}$.

5. Wealth strengthens financial wellbeing (from Equation (5)):

$$F_{t}=f_{F}\left({{OF}_{t;}SF}_{t}\right)$$

with

$$f_{F}^{\prime }\left({OF}_{t}\right)>0;$$

therefore

$$\frac{\partial f_{F}}{\partial {OF}_{t;}}>0$$

In summary:

$$C_{OF,t}↑\Rightarrow P_{t+1}↑\Rightarrow higher\;likelihood\;that\;H_{t+1}>0\Rightarrow {Y(P}_{t})>0\Rightarrow {OF}_{t+1}↑\Rightarrow F_{t+1}↑$$

An increase in health spending leads to a higher likelihood of an individual earning labour income for longer, which increases the time that they can accumulate financial wealth (objective wellbeing), which improves his/her financial wellbeing.

**Proposition** **2.** *The simultaneous improvement in physical and financial wellbeing for all t also increases the integrated wellbeing of the individual at each point in time (from Equation (4)).*

$${IW}_{t}=F_{t}^{\theta_{F}}P_{t}^{\theta_{P}}$$

$$\theta_{F},\theta_{P}>0.$$

Therefore, if $F_{t}^{A}\geq F_{t}^{B}\;and\;P_{t}^{A}\geq P_{t}^{B}$, then

$${{IW}_{t}}^{A}={\left(F_{t}^{A}\right)}^{\theta_{F}}\;{\left(P_{t}^{A}\right)}^{\theta_{P}}\geq \;{\left(F_{t}^{B}\right)}^{\theta_{F}}\;{\left(P_{t}^{B}\right)}^{\theta_{P}}={{IW}_{t}}^{B}$$

**Proposition** **3.** *Within the health span, higher physical wellbeing raises labour income, which can substitute for interest income. Therefore, for a fixed target path of objective wealth, improved physical wellbeing allows the individual to sustain the same wealth with a lower required rate of return, ceteris paribus (from Equation (12)).*

$${OF}_{t}={{OF}_{t-1}+r}_{t-1}OF_{t-1}+{Y(P}_{t-1})+I_{OF,t}-C_{OF,t}\mathrm{if}\;H_{t-1}>0$$

Rearranging Equation (12) to solve for the return $\left(r_{t-1}\right)$ required to achieve a certain level of objective wealth (${OF}_{t}$):

$$r_{t-1}\;=\;\frac{{OF}_{t}-OF_{t-1}-{Y(P}_{t-1})-\;I_{OF,t}+C_{OF,t}}{{OF}_{t-1}}$$

Assuming ${OF}_{t-1}>0$ and differentiating with respect to $P_{t-1}:$

$$\frac{\partial r_{t-1}}{\partial P_{t-1}}=-\frac{Y^{\prime }(P_{t-1})}{{OF}_{t-1}}$$

Within health span, $Y^{\prime }(P_{t-1})>0$ (higher physical wellbeing raises labour income).

Therefore:

$$\frac{\partial r_{t-1}}{\partial P_{t-1}}<0$$

The second derivative of this relationship is, however, positive (assuming diminishing marginal returns on health spending), suggesting a diminishing marginal impact of health on required return.

**Figure 1:**
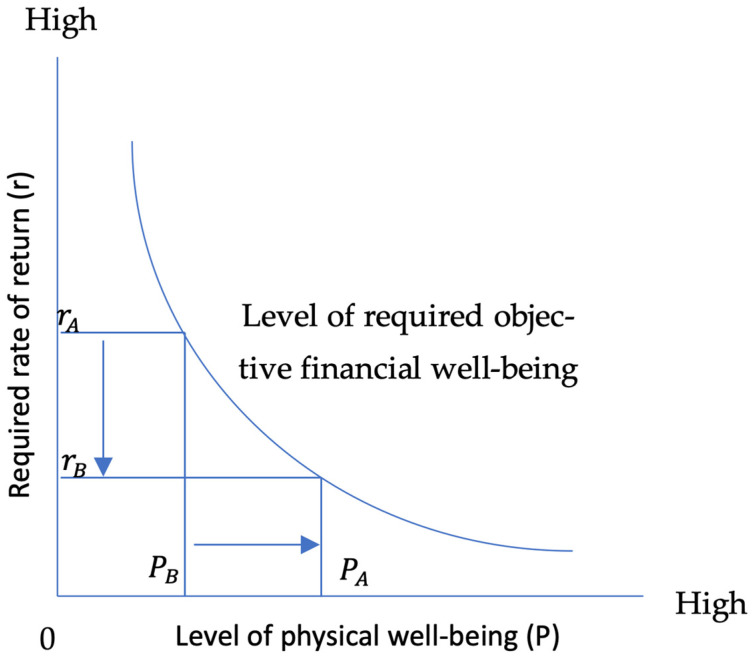
The relationship between the required rate of return (r) on financial wellbeing and the level of physical wellbeing (P).

The relationship between the required rate of return on objective financial and physical wellbeing can be demonstrated in the graph below where the required rate of return $r_{A}$ is associated with a level of physical wellbeing $P_{B}$. Lifting the level of physical wellbeing to $P_{A}$ is associated with a lower level of required return $r_{B}$ for the same level of required objective financial wellbeing (Figure 1).

## 6. Discussion and Implications

The growing body of research on human wellbeing indicates that its constituent domains do not operate independently but are dynamically and contextually interdependent. These interactions generate complex feedback loops that need to be considered when analysing individual domains as well as wellbeing in its totality. The integrated nature of wellbeing implies that optimisation within one domain may be achieved indirectly through investment in another. Moreover, optimising wellbeing across both the short and long term requires deliberate balancing of scarce resources across domains and across the lifespan.

To formalise these interdependencies, we model integrated wellbeing using a Cobb–Douglas function combined with domain-specific investment functions and explicit resource constraints. These constraints reflect not only economic scarcity but also an individual’s willingness and ability to sacrifice gains in one domain to enhance another in pursuit of long-term integrated wellbeing. This systemic structure allows us to examine how trade-offs and complementarities unfold over time.

Applying the model to financial and physical wellbeing demonstrates that optimisation strategies differ substantially from traditional domain-specific approaches. Conventional financial advice typically emphasises return maximisation through improved asset selection or higher-yield opportunities. However, our model indicates that financial wellbeing can be enhanced indirectly through improvements in physical wellbeing, without increasing expected financial return rates. In other words, strengthening physical health expands productive capacity and financial resilience, thereby shifting the financial optimisation frontier itself.

Importantly, the model also demonstrates that a narrow focus on financial return rates leads to diminishing marginal gains within that domain. In contrast, cross-domain investment in physical wellbeing raises integrated wellbeing while simultaneously mitigating diminishing marginal returns, because both domains improve concurrently. This systemic interaction produces upward adjustments in overall wellbeing that are not attainable through siloed optimisation strategies.

This approach challenges conventional financial planning paradigms, which tend to isolate financial objectives from broader human development domains. By embedding financial wellbeing within an integrated system, the model offers a foundation for rethinking optimisation across the lifespan. At present, the framework remains theoretical and requires empirical validation, but it establishes a formal structure for analysing cross-domain spillovers and intertemporal trade-offs.

Theoretically, this framework advances systemic perspectives within positive psychology by operationalising integrated flourishing in a formal economic model. By representing physical and financial wellbeing as interdependent components of an integrated utility structure, the research aligns with third-wave positive psychology’s emphasis on complexity and contextual embeddedness. The model extends multidimensional wellbeing theory by demonstrating mathematically how cross-domain complementarities can offset diminishing marginal returns within single domains. In doing so, it bridges positive psychology, health economics, and lifespan optimisation theory, offering a structured account of how upward spirals of resource development may occur across domains.

The framework also incorporates applied implications on various fronts. From a financial planning perspective, the findings suggest that health-related expenditures should be conceptualised as strategic capital investments rather than consumption costs. Integrating preventive health behaviours, resilience-building, and strengths-based interventions into lifecycle planning may enhance long-term financial stability beyond traditional return-focused strategies. For health policy, the results support upstream investment in psychological and physical resource development, consistent with evidence linking optimism, purpose, and other psychological assets to improved health behaviours and longevity. More broadly, wellbeing interventions may benefit from cross-domain alignment in which physical health promotion, behavioural adherence, and financial capability development are coordinated to produce reinforcing systemic gains rather than isolated improvements.

In practice, this framework can be applied by multiple stakeholders to aid decision-making in the allocation of resources. Financial planners and wealth managers can incorporate physical health indicators and behavioural factors into client assessments, reframing health investments as integral to financial strategy and long-term portfolio resilience. Healthcare providers and allied health professionals can utilise the model to design interventions that explicitly consider patients’ financial and behavioural contexts, thereby improving adherence and long-term outcomes. Employers and organisational leaders may apply the framework within workplace wellness programmes by aligning physical health initiatives with financial wellbeing support, such as incentives, education, and behavioural nudges. Policymakers can leverage these insights to design integrated public health and financial resilience strategies that prioritise preventive care and cross-sector collaboration. At the individual level, the model offers a decision-making heuristic for allocating time, effort, and financial resources across domains to optimise overall wellbeing across the lifespan. In this way, the theoretical model translates into practice by providing a structured lens through which coordinated, cross-domain interventions can be designed, implemented, and evaluated.

## 7. Limitations and Future Work

A key limitation of the current state is that it requires empirical estimates. The application also simplifies integrated wellbeing into two domains, omitting other domains that may be harder to quantify (e.g., vocational, spiritual, relational, mental and social dimensions, to name but a few).

Future work that could build on the framework includes empirically estimating behavioural parameters and domain elasticities within integrated wellbeing functions to quantify cross-domain spillover magnitudes. The model should be expanded to incorporate additional generic domains to better reflect multidimensional flourishing in specified and varied contexts. Incorporating stochastic elements would allow the framework to account for uncertainty and exogenous shocks affecting domain trajectories. Longitudinal and mixed-methods research designs could test whether integrated investment strategies generate measurable upward spirals over time. As empirical knowledge of contextualised domain interactions improves, the model should be refined.

## 8. Conclusions

The findings demonstrate that human wellbeing is a dynamically interdependent system in which cross-domain interactions shape integrated wellbeing outcomes across the lifespan. Consistent with contemporary positive psychology’s systemic and strengths-based orientation, the results show that developing capacity in one domain can support and enhance another, mitigating constraints without direct optimisation. By illustrating how improvements in one dimension of wellbeing enhance another, the model reflects resource-building processes and upward spirals central to positive psychology’s understanding of flourishing.

The study extends positive psychology by providing a quantitative, systems-level framework for analysing and optimising multidimensional wellbeing. Rather than relying on narrow strategies constrained by diminishing returns, coordinated cross-domain investments generate reinforcing gains. Practically, this framework can inform financial planning, healthcare, and policy by enabling integrated, preventative, and strengths-based interventions.

Although theoretical, the model provides a foundation for empirical validation and advancing systemic approaches to sustainable, whole-entity flourishing.

## Figures and Tables

**Table 1:** Summary description of variables included in the integrated wellbeing system.

Variable	Description
${IW}_{t}$	Integrated wellbeing of an individual in period t
$x_{i,t}$	Represents wellbeing domain i in period t
$\theta_{i}$	Elasticity of wellbeing domain i on the individual’s integrated wellbeing
$I_{i,t}$	The total investment in the development of wellbeing domain *x_i_* in period t
$R_{t}$	Total resources that an individual has available to expend on the maintenance and development of his/her wellbeing domains in period t
$C_{it}$	Represents the resources expended on developing wellbeing domain i
$\delta_{i}$	Represents the rate of decline of wellbeing domain i

## Data Availability

The original contributions presented in this study are included in the article. Further inquiries can be directed to the corresponding author.

## Abbreviations

The following abbreviations are used in this manuscript:

IW	Integrated Wellbeing
WB	Wellbeing
